# Post-monsoon waterlogging-associated upsurge of cholera cases in and around Kolkata metropolis, 2015

**DOI:** 10.1017/S0950268819000529

**Published:** 2019-03-29

**Authors:** Asish K. Mukhopadhyay, Alok K. Deb, Goutam Chowdhury, Falguni Debnath, Prosenjit Samanta, Rudra Narayan Saha, Byomkesh Manna, Mihir K. Bhattacharya, Dharitri Datta, Keinosuke Okamoto, Uchhal K. Bhadra, Shanta Dutta

**Affiliations:** 1 Division of Bacteriology, National Institute of Cholera & Enteric Diseases, Kolkata, India; 2 Division of Epidemiology, National Institute of Cholera & Enteric Diseases, Kolkata, India; 3 Division of Data management, National Institute of Cholera & Enteric Diseases, Kolkata, India; 4 Division of Clinical Medicine, National Institute of Cholera & Enteric Diseases, Kolkata, India; 5 Collaborative Research Center of Okayama University for Infectious Diseases at NICED, Kolkata, India; 6 ID&BG Hospital, Kolkata, India

**Keywords:** Cholera, diarrhoea, *Vibrio cholerae*

## Abstract

The Infectious Diseases and Beliaghata General Hospital, Kolkata, India witnessed a sudden increase in admissions of diarrhoea cases during the first 2 weeks of August 2015 following heavy rainfall. This prompted us to investigate the event. Cases were recruited through hospital-based surveillance along with the collection of socio-demographic characteristics and clinical profile using a structured questionnaire. Stool specimens were tested at bacteriological laboratory of the National Institute of Cholera and Enteric Diseases (NICED), Kolkata. Admission of 3003 diarrhoea cases, clearly indicated occurrence of outbreak in Kolkata municipal area as it was more than two standard deviation of the mean number (911; s.d. = 111) of diarrhoea admissions during the same period in previous 7 years. Out of 164 recruited cases, 25% were under-5 children. Organisms were isolated from 80 (49%) stool specimens. *Vibrio cholerae* O1 was isolated from 50 patients. Twenty-eight patients had this organism as the sole pathogen. Among 14 infants, five had cholera. All *V. cholerae* O1 isolates were resistant to nalidixic acid, followed by co-trimoxazole (96%), streptomycin (92%), but sensitive to fluroquinolones. We confirmed the occurrence of a cholera outbreak in Kolkata during August 2015 due to *V. cholerae* O1 infection, where infants were affected.

## Introduction

Cholera is a severe form of waterborne acute dehydrating diarrhoeal disease which is well known for its epidemic and pandemic potentials [1, 2]. Cholera continues to be a major public health problem, particularly in developing countries where access to potable drinking water and hygienic sanitation [3, 4] remains inadequate. Toxigenic strains of *Vibrio cholerae* serogroups O1 and O139 are the causative agents of cholera, and exhibited several changing patterns in the biotype as well as in drug resistance for better survival and infection ability [5, 6]. In 2014, 42 countries reported to the World Health Organization (WHO) a total of 190 549 cholera cases with 2231 deaths, resulting in an overall case fatality rate (CFR) of 1.17%. About 55% of all reported cases originated from Africa followed by Asia. In India, 4031 cholera cases with 21 deaths (CFR, 0.52%) were reported from 12 States and most of these cases (49%) were reported from West Bengal [7].

Cholera exists as a seasonal disease in India especially in Kolkata, the capital city of the state of West Bengal located in the Gangetic delta and has been greeted as the ‘homeland of cholera’ [8–10]. Cholera cases occur round the year in this city with different magnitudes. This disease becomes more pronounced in those areas where there is post-monsoon water logging or overflow of drains contaminating surface water bodies due to inadequate drainage system [11].

During July 2015, there was a heavy rainfall in Kolkata which led to overflow of many drains and water logging at certain parts of this city. In the first week of August, Infectious Diseases and Beliaghata General (ID&BG) Hospital of eastern Kolkata witnessed a huge increase in the number of diarrhoea patients requiring admission for treatment. The National Institute of Cholera and Enteric Diseases (NICED), Kolkata, has been conducting diarrhoeal disease surveillance in this hospital for almost past two decades, where systematically every fifth hospitalised diarrhoea case on two selected days in each week is recruited [12]. In response to this flood situation, scientists at NICED analysed surveillance data of a subset of the diarrhoea patients admitted to ID&BG Hospital from Kolkata Municipal Corporation area during July–August 2015. The investigation was undertaken to confirm existence of any diarrhoea disease outbreak, to recommend appropriate preventive and control measures, to identify the aetiological agents of outbreak, to determine the antimicrobial susceptibility of the isolated organism against a range of antimicrobial agents and to determine the genetic relatedness of the isolated strains during this period.

## Materials and methods

### Epidemiological analysis to understand the existence of a diarrhoeal outbreak

Information on selected demographic, socio-economic and clinical profiles was collected by trained NICED staff using the structured questionnaire from the patients or the parent caregivers in case of children through the routine surveillance of NICED. The clinical assessment was made according to WHO guidelines [13]. Data were checked manually and subsequently entered in the surveillance-specific database designed at NICED. Analyses were performed using the statistical software STATA SE ver. 8.2 (Stata Corp, Texas, USA).

The mean of hospital admissions from Kolkata metropolis area during first 2 weeks of August of 2007–2014 were also compared to understand the magnitude of the situation in 2015.

### Laboratory investigation

Stool specimens from the patients were collected using rectal catheters and were transported within 2 h of collection to the laboratory at NICED for further processing and testing as described below.

### Stool culture

The samples were processed for isolation of common enteric pathogens such as *Vibrio* spp., *Salmonella* spp., *Shigella* spp., diarrhoeagenic *Escherichia coli* and *Campylobacter* spp. [14]. Alkaline peptone water was used for the enrichment of *V. cholerae*, whereas Gram-negative broth (Difco, BD, USA) was used for the enrichment of *Salmonella* spp. and *Shigella* spp. Enrichments were sub-cultured on thiosulfate-citrate-bile salts-sucrose (TCBS) (Difco) for *V. cholerae* and Hektoen enteric agar (HEA) and xylose lysine deoxycholate agar (XLD agar) (Difco) for *Salmonella* spp. and *Shigella* spp. All plates were incubated overnight at 37 °C. The yellow-coloured colonies on TCBS media for suspected *V. cholerae* were subjected to standard biochemical tests, including sugar fermentation in triple sugar iron agar, and oxidase production. Detected *V. cholerae* were identified to belong to O1 serogroup through slide agglutination test using antisera kit (Difco). Suspected colonies from HEA and XLD agar for *Salmonella* spp. and *Shigella* spp. were further confirmed by biochemical and serotyping using commercially available antisera (Denka-Seiken, Tokyo, Japan). Stool specimens were also inoculated onto MacConkey agar (Difco) plates and incubated overnight for identification of *E. coli*. Lactose fermenting colonies were further tested in pathogroup-specific PCR assays for identification of diarrhoeagenic *E. coli*. Stool specimens were also inoculated onto blood agar plates and kept under microaerophilic conditions for the presence of typical translucent colonies for presumptive identification of *Campylobacter* spp., which was subsequently confirmed by PCR assay.

### Antimicrobial susceptibility testing

Antimicrobial susceptibility testing was performed using the disk diffusion method with commercially available disks. The disks used included ampicillin (AMP_10_), ceftriaxone (CRO_30_), chloramphenicol (C_30_), erythromycin (E_15_), gentamicin (GM_10_), nalidixic acid (NA_30_), ciprofloxacin (CIP_5_), ofloxacin (OFX_5_), norfloxacin (NOR_10_), meropenem (MEM_30_), streptomycin (STR_10_), azithromycin (AZM_15_), tetracycline (TET_30_), trimethoprim/sulfamethoxazole (SXT_1.25/23.75_), neomycin (N_30_) (Becton Dickinson, Sparks Glencoe, MD, USA) in accordance with the criteria recommended by Clinical and Laboratory Standards Institute [15]. According to CLSI guidelines, breakpoints for *Enterobacteriaceae* were used to determine antimicrobial susceptibility. *Escherichia coli* ATCC 25922 was used as a quality control strain.

### PCR analysis

All the clinical isolates of *V. cholerae* were grown on Luria–Bertani (LB) Agar (Difco) plates. DNA extractions were carried out by phenol–chloroform extraction method [16] and were diluted to desired concentration for using as a template during PCR assay. Allele-specific PCR-based assay for the virulence-associated genes namely, cholera toxin subunit B (*ctxB*), toxin co-regulated pilus A (*tcpA*) and repeats-in-toxin A (*rtxA*) was performed using primer sets constructed by exploiting the single nucleotide polymorphism. DMAMA-PCR was done for identifying *ctxB* genotype 7 found in Haitian variant strains using primers ctxB-F3/Rv-Cla and for identifying genotype 1 found in classical biotype strains using ctxB-F4/Rv-Cla [17]. Another allele-specific PCR was carried out with the primer sets tcpA F1/El-Rev for El Tor type and tcpA F'2/El-Rev for variant type *tcpA* [18]. Further, different alleles of *rtxA* were identified with one common forward (rtxAF) and two reverse primers (rtxA-R1 and rtxA-R2) specific for El Tor and *rtxA*-null mutant, respectively [19, 20].

### Pulsed-field gel electrophoresis

Pulsed-field gel electrophoresis (PFGE) was performed with the representative *V. cholerae* strains isolated during this period according to the Pulse Net protocol for *V. cholerae* [21]. DNA from *Salmonella enterica* serotype Braenderup strain H9812 was subjected to restriction digestion with *XbaI* (Takara, Japan) and employed as the universal size standard. The DNA of test strains was digested with *NotI* and subjected to electrophoresis. The gel was then stained for 30 min using ethidium bromide solution (5 µg/ml), followed by rinsing several times in distilled water. The band pattern was observed under UV illumination (BioRad, Hercules, CA, USA). The DNA fingerprint patterns of the test strains and the reference strains of *V. cholerae* were analysed using the computer software package BioNumerics (Applied Maths, Kortrijk, Belgium). The fingerprint patterns were also subjected to typing based on banding similarity and dissimilarity using the Dice similarity coefficient. The cluster analysis was performed based on the single-linkage method. Finally, the results were graphically represented as dendrograms.

## Result

### Confirmation of existence of an ongoing diarrhoeal outbreak

**Fig. 1.**
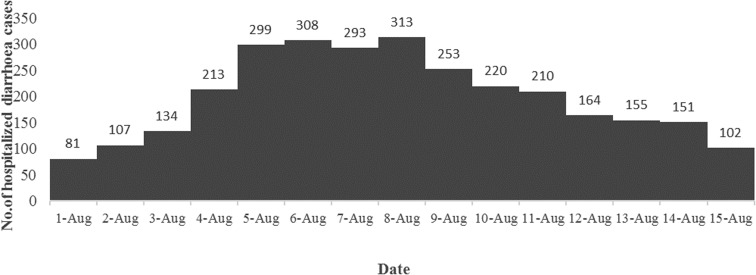
Distribution of the number of diarrhoea cases admitted to the ID&BG Hospital, Kolkata during 1–15 August 2015.

A total of 3003 diarrhoea cases were admitted to ID&BG Hospital during 1–15 August 2015 (Fig. 1), which was more than three times of the mean number of diarrhoea cases (911; s.d. = 111) admitted to the same hospital during the same period in the previous 7 years (2007–2014) confirming the occurrence of a diarrhoea outbreak in Kolkata municipal area during that period.

### Descriptive epidemiology

Among the total 3003 diarrhoea admitted cases during this period, 164 patients of all age groups and genders were recruited under the surveillance system following the sampling procedure stated earlier. All the age groups were affected in this outbreak (minimum age: 5 months; maximum: 99 years) and around a quarter of the cases (42 of 164) were below 5 years of age. Male patients predominated over females. Less than a fifth (~20%) of the patients had more than secondary education, which differed significantly between the two genders (male: 26.6%; female: 9.1%; *P* = 0.008). The average monthly income of the families was around Rs. 9000 (~US$135). Most of the families (>95%) used tap water for drinking purpose (Table 1).

**Table 1.** Socio-demographic characteristics of the diarrhoea cases

Socio-demographic characteristics	Distribution (*N* = 164)
Age (years)	
Mean ± s.d.	28.3 ± 22.5
Median	26.0
Age groups (years)	
<1	14 (8.5%)
1 to <5	28 (17.1%)
5 to <18	17 (10.4%)
18 to <30	27 (16.5%)
30 to <50	46 (28.1%)
⩾50	32 (19.5%)
Gender	
Male	92 (56.1%)
Female	72 (43.9%)
No. of members in the family	
Mean ± s.d.	5.4 ± 1.7
Monthly family income (Rs.)	
Mean ± s.d.	9062 ± 2504
Up to Rs. 7500	41 (25.0%)
Rs. 7501–9000	55 (33.5%)
Rs. 9001–10 000	41 (25.0%)
>Rs. 10 000	27 (16.5%)
Education status (if aged ⩾7 years; *n* = 119)	
Illiterate	16 (13.5%)
Primary (grades 1–4)	11 (9.2%)
Middle (grades 5–8)	40 (33.6%)
Secondary (grades 9–10)	30 (25.2%)
>Secondary	22 (18.5%)
Occupation of primary earning member in the family	
Unskilled labour	33 (20.1%)
Skilled worker	43 (26.2%)
Service (Govt./private)	61 (37.2%)
Business	27 (16.5%)
House type	
Pucca	131 (79.9%)
Semi-pucca	30 (18.3%)
Mud house	3 (1.8%)
Source of drinking water	
Tap	157 (95.7%)
Tube well	7 (4.3%)

### Spatial distribution of the diarrhoea cases

**Fig. 2.**
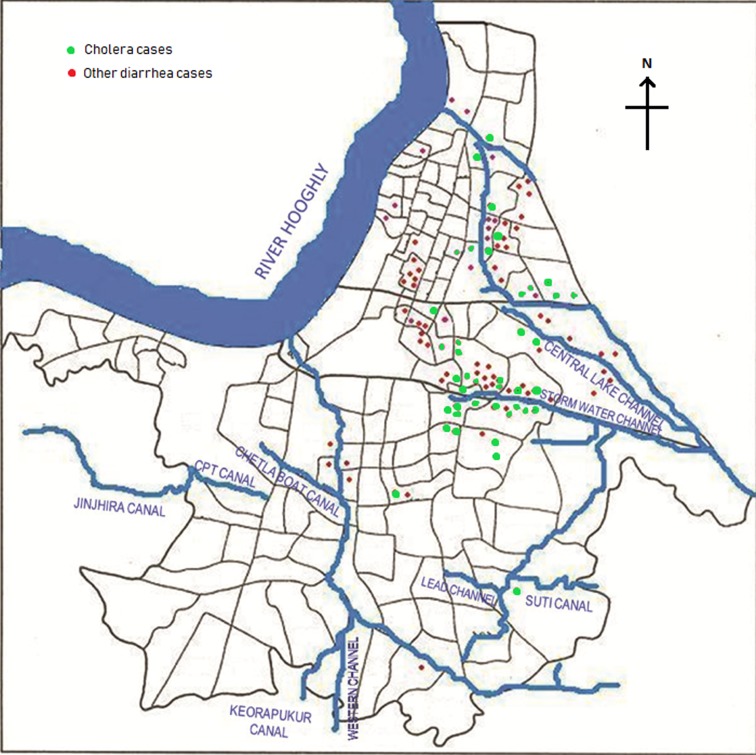
Spot map of Kolkata Municipal Corporation area showing spatial distribution of diarrhoea cases during the outbreak. Green dots indicate culture-confirmed cholera cases whereas the red dots denote diarrhoea with other pathogens.

Of the total 164 diarrhoea cases recruited under the surveillance, 129 (79%) were residents of the Kolkata Municipal Corporation (KMC) area and the remaining patients came from neighbouring districts of Kolkata (mostly from north and south 24-Parganas). The KMC area is spread over 205 km^2^, and with a population close to 5 million residing in 144 wards (smallest administrative units of KMC), is dense (24 760 persons/km^2^) (https://www.kmcgov.in/KMCPortal/jsp/KMCPortalHome1.jsp). The river Hooghly runs along its western boundary and several canals traverse the municipal area for draining flood and storm waters into the river. Heavy rains and/or blockages of drainage systems for various reasons often lead water-logging in the affected areas in monsoon season, leading to contamination of drinking water and increasing the risk of waterborne diseases, especially gastro-intestinal infections. We plotted the diarrhoea cases occurring within the KMC area onto a map of that area to understand their spatial distribution and observed that most of the recruited 164 diarrhoea cases occurred either in areas along the canal which runs through Kolkata metropolis or within some selective geographic locations (Fig. 2). Distribution of cholera cases also revealed the similar patterns.

### Clinical characteristics of the diarrhoea cases

On an average, the patients attended the ID&BG Hospital more than a day after the onset of diarrhoea. About 70% of them had already visited a health care provider before attending the hospital – the commonest provider being an allopathic medical practitioner (54%), followed by a pharmacy outlet (25%) and a government health care facility (17%). Ninety-five per cent of the patients presented with watery diarrhoea, with a mean stool frequency of nine per day. All the recruited patients received intravenous fluid on admission and interestingly a majority of them received ORS or home available fluid for diarrhoea management even prior hospital admission. Forty-two per cent cases had received some antimicrobial drug(s) before admission, 30% could not tell about their intake of any antimicrobial agent. Except one patient with ‘severe’ dehydration, all others presented with ‘some’ degree of dehydration on admission (Table 2). One of these patients (a 52-year-old female with diarrhoea and vomiting) died in the hospital due to cardiovascular complication; remaining patients were improved during the hospital stay and discharged after complete recovery.

**Table 2.** Clinical characteristics of the diarrhoea cases

Clinical characteristic	Distribution (*N* = 164)
Visited another care provider before attending this hospital	
Yes	114 (69.5%)
No	50 (30.5%)
If *Yes*, type of care provider (*n* = 114)	
Pharmacy	29 (25.4%)
Govt. health facility	19 (16.7%)
Private health facility	3 (2.6%)
Private doctor (allopathic)	62 (54.4%)
Private doctor (homeopathic)	1 (0.9%)
Duration of diarrhoea before admission (hours)	
Mean ± s.d.	28.9 ± 17.9
Stool consistency	
Watery	156 (95.1%)
Loose	3 (1.8%)
Bloody	2 (1.2%)
Bloody and mucoid	3 (1.8%)
No. of stool passed before admission	
Mean ± s.d.	8.6 ± 2.5
Median	9
Other symptoms associated with diarrhoea	
Vomiting	135 (82.3%)
Fever	42 (25.6%)
Abdominal pain	83 (50.6%)
Received antimicrobials before admission	
Yes	69 (42.1%)
No	47 (28.7%)
Not sure	48 (29.3%)
Received ORS before admission	
Yes	131 (79.9%)
No	33 (20.1%)
Received home available fluid before admission	
Yes	99 (60.4%)
No	65 (39.6%)
Dehydration status on admission	
Severe	1 (0.6%)
Some	163 (99.4%)
Duration of hospital stay (hours)	
Mean ± s.d.	31.5 ± 22.8
Median	27

### Laboratory investigation

Stool specimens were collected from all recruited diarrhoea patients and organisms were isolated from 80 (49%, 80/164) stool specimens. Out of the 80 microbiologically confirmed specimens, the most commonly isolated organism was *V. cholerae* O1 (50). From stool specimen of 28 patients, *V. cholerae* was isolated as the sole pathogen, whereas in 22 stool specimens, the organism was isolated along with other pathogen(s). Serological confirmation revealed that 49 belonged to O1, Ogawa and only one belonged to Inaba serotype. Patients aged between 18 and <30 years were mostly affected with cholera (13 of 27; 48%). Interestingly among 14 diarrhoea infants, five had cholera with *V. cholerae* O1 infection. No significant difference was found between male and female cases (*M* = 35%, *F* = 25%; *P* = 0.177) in isolation rate of *V. cholerae* organism.

Apart from diarrhoea cases recruited under the surveillance, forty additional stool specimens were collected from diarrhoea cases (not included in the surveillance) attending the hospital during the same period for better understanding of the aetiology of this outbreak and processed using similar procedures. However, the socio-demographic and clinical information was not collected for these cases.

**Fig. 3.**
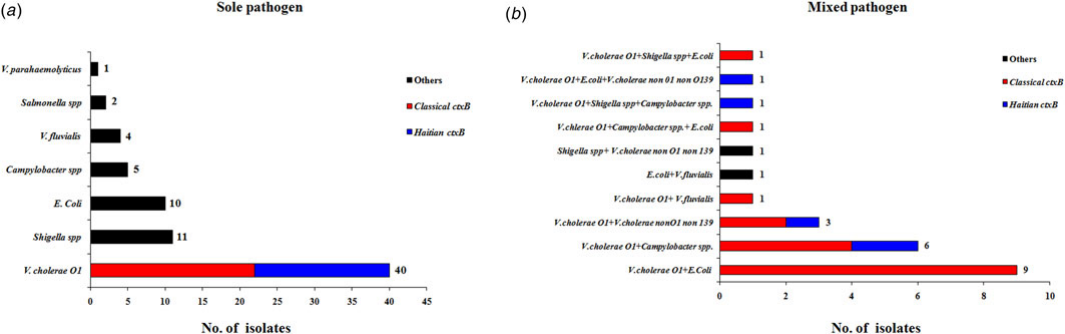
Distribution of different enteric bacterial pathogens isolated from the stool specimens (*N* = 204) during 1–15 August 2015. *V. cholerae* strains were marked with two different colour codes based on their *ctxB* genetic status. (a) Indicates the distribution of sole pathogens in diarrhoeal patients and (b) depicts the number of patients infected with different set of mixed pathogens.

The results obtained from testing a total of 204 stool specimen were as follows: microbiological analysis revealed that 63 (31%) were positive for *V. cholerae* O1, 23 (11%) for diarrhoeagenic *E. coli*, 14 (7%) for *Shigella* spp. and 13 (6%) with *Campylobacter* spp. In addition, six (3%) samples each were positive for *V. fluvialis*, five (2%) for *V. cholerae* non-O1, non-O139, two (1%) for *Salmonella* spp. and one (0.4%) was positive for *V. parahaemolyticus*. The distribution of total pathogens isolated from the outbreak samples is shown in Figure 3. A total of 98 stool specimens were positive for at least one pathogen and 73 samples were positive for sole pathogen. *V. cholerae* O1 was isolated as a sole pathogen from 40 (20%) of 204 stool samples screened.

**Fig. 4.**
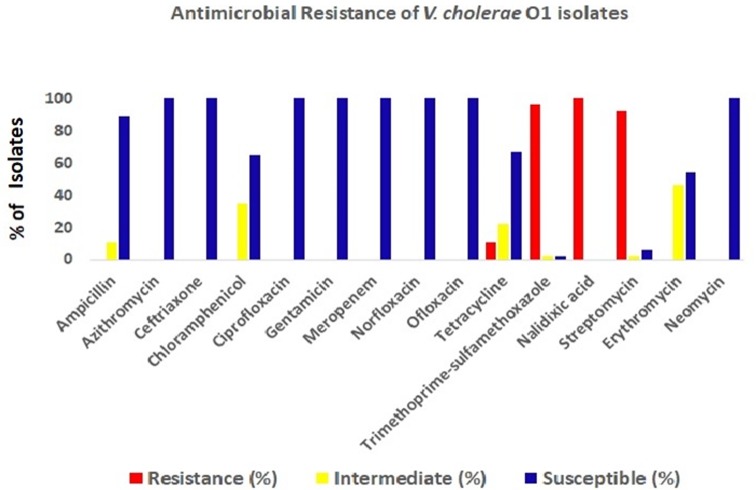
Antibiotic-resistance profile of the *V. cholerae* isolates (*n* = 34) during the study period. Different colour codes for sensitive, resistance and intermediate level of resistance were used. Red, blue and yellow colour codes indicate resistant, sensitive and intermediate phenotypes, respectively.

The antimicrobial disc diffusion test revealed that all the *V. cholerae* O1 isolates were resistant to nalidixic acid (100%), followed by co-trimoxazole (96%), streptomycin (92%) and tetracyclin (11%). But all isolates were sensitive to ciprofloxacin, ofloxacin, norfloxacin, meropenem, neomycin, gentamicin and azithromycin (Fig. 4).

### Genetic features of the *V. cholerae* isolates

All the *V. cholerae* O1 isolates were tested by already established DMAMA PCR assay using two allele-specific forward primers and one common *ctxB* reverse primer for discriminating the classical, El Tor and Haitian type *ctxB* alleles. Surprisingly, around 65% of the *V. cholerae* strains yielded amplicons with *ctxB* classical-specific PCR primers and the rest of the isolates were positive for *ctxB* Haitian-specific PCR primer set indicating reappearance of classical *ctxB* (*ctxB*1) after 3 years of absence. Prior to this cholera upsurge, all the *V. cholerae* strains isolated at Kolkata contained the Haitian variant *ctxB (ctxB7*) from the middle of 2012 to first half of 2015. All the tested strains produced amplicons for Haitian-specific *tcpA* (*tcpA*^CIRS^) but not with the El Tor-specific *tcpA*. Interestingly, all the strains, which yielded amplicons for classical *ctxB* (*ctxB*1), were positive for El Tor-specific *rtxA*. On the other hand, the remaining strains, which produced amplicons for Haitian *ctxB* (*ctxB*7), were positive for Haitian-specific *rtxA*. So, based on the analysis of *ctxB*, *tcpA* and *rtxA*, the isolated strains during these 2 weeks period can be divided in two different genetic combinations namely, strains with Classical *ctxB* (*ctxB*1)_Haitian *tcpA* (*tcpA*^CIRS^)_El Tor *rtxA* and Haitian *ctxB* (*ctxB*7)_*tcpA*^CIRS^_variant *rtxA* (Table 3). To determine the genetic relatedness among *V. cholerae* strains, 14 representative strains were compared by using PFGE analysis. Dendrogram analysis using Bionumeric software (Applied Maths, Belgium) showed overall similarity of more than 95% and five pulsotype patterns of PFGE were obtained where predominant pulsotypes are P1 and P4. Interestingly, all the P1 pulsotype strains carried Haitian *ctxB* (*ctxB*7)_*tcpA*^CIRS^_variant *rtxA*, whereas strains containing P2-P5 pulsotypes had Classical *ctxB* (*ctxB*1)_Haitian *tcpA* (*tcpA*^CIRS^)_El Tor *rtxA.* But the isolation of *V. cholerae* strains with two genetic types from the affected population did not show any geographic clustering.

**Table 3.** Genetic background and mode of infection of *V. cholerae* O1 strains isolated in this study

Total No. of sample screened	Total No. of samples positive for *V. cholerae* O1	Sole infection with *V. cholerae* O1	Mixed infection along with *V. cholerae* O1 (*)	No. of *V. cholerae* containing classical *ctxB*, Haitian *tcpA*, El Tor *rtxA*	No. of *V. cholerae* containing Haitian *ctxB*, Haitian *tcpA*, Haitian *rtxA*
204	63	40	23	41	22

*Mixed infection along with *V. cholerae* O1 includes other pathogens which have been depicted in Figure 3.

## Discussion

In this study, *V. cholerae* was the most commonly isolated pathogen among the collected stool specimens. Little less than half of the recruited diarrhoea patients received antimicrobials before hospital admission, and interestingly, almost one-third of the recruited cases could not give any information on antimicrobial consumption history which possibly explains the absence of any pathogen in 84 collected stool specimens. Based on all these facts, we confirmed that there was an increased number of cholera cases during first 2 weeks of August 2015 in Kolkata metropolis area alongside the storm water channel and central lake channel which runs through east Kolkata. This might have occurred by contamination of drinking-water sources due to overflowing of canals/drains during the heavy rainfall in July 2015.

**Fig. 5.**
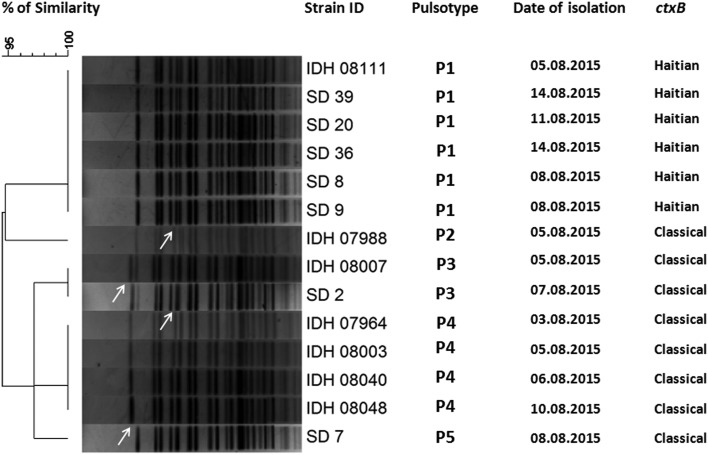
Pulsed-field gel electrophoresis analysis of *NotI*-digested genomic DNA of *V. cholerae* strains (*n* = 14) isolated from diarrhoeal faecal samples in Kolkata, India during August 2015. The image of the gel was analysed using BioNumerics version 4.0 software (Applied Maths) based on the single-linkage method to generate the dendrogram. Per cent similarity was shown at the left-hand side. PFGE analysis revealed that all the isolates shared around 95% similarity along with five pulsotype patterns but P1 pulsotype strains carried Haitian *ctxB* whereas P2-P5 pulsotype strains contained classical *ctxB*.

Usually cholera epidemics are linked to monsoon followed by contamination of drinking water by sewage or polluted water bodies. Additionally, lack of proper distribution of safe drinking water, healthy hygiene and sanitation increases the burden in endemic areas [22]. Though in this study, majority of the recruited diarrhoea cases used municipal tap water supply as a drinking-water source, but instances of leakage in municipal pipeline water supply in West Bengal are common occurrence. Hence, chance of occurrence of waterborne disease outbreaks like cholera increases during monsoon or flood situations as drinking-water sources get easily contaminated by overflowing of sewerage drains [23–25]. To avert such occurrences, piped water supply had been reinforced as improved drinking-water source, by Millennium development goal. Moreover, sustainable development goal 6 also addresses the issues of drinking water, sanitation and hygiene (WaSH), along with quality and sustainability of waterbodies worldwide [26] as periodic vigilance of, drainage and water supply system are the key to ensure supply of safe drinking water during natural calamities.

*V. cholerae* O1 was isolated from 31% of the stool specimens collected and processed from hospitalised patients. Genetic analysis of the *V. cholerae* strains revealed the reappearance of classical *ctxB* in Kolkata during 2015. Interestingly, 65% strains had genetic combination of classical *ctxB*, Haitian *tcpA* and El Tor *rtxA*. PFGE analysis of the representative *V. cholerae* strains yielded more than 95% similarity indicating the close genetic relatedness among these strains.

Cholera outbreaks due to multiple antibiotic-resistant *V. cholerae* have emerged as a major public health problem in last two decades [27]. In our study, multiple antibiotic-resistant *V.cholerae* isolates were recovered which was a matter of concern for a physician to treat the disease. In the present government-run routine disease surveillance system, there is no scope for keeping an account of the antibiotic sensitivity pattern of diarrhoea-causing pathogens, but it is immensely important as antimicrobial resistance has become a global public health threat.

This investigation had few limitations. Since, diarrhoea patients were recruited through ongoing hospital-based diseasesurveillance of ICMR – NICED, the actual number of affected people could not be ascertained, but still we could identify the situation when the cholera cases increased in Kolkata Metropolitan area and informed the local municipal authority about that. Second, we did not carry out any analytical epidemiological study in the field due to logistic constraints, but still from the literature review, it was evident that, contamination of water bodies was common in monsoon and post-monsoon period in Kolkata. We conclude that this outbreak in and around Kolkata during August 2015 was due to infection predominantly with *V. cholerae* O1, Ogawa and partially due to the other pathogens like *Shigella*, *E. coli* and *Campylobacter*. Isolation of multiple pathogens in the outbreak suggests that either remaining flood waters or the water supply are likely to be the source of the infections. However, confirmation would require testing the water supply or the remaining flood waters, which were not done during the episode.

## Recommendations

Based on the findings, the local municipal corporation was alerted about the situation. Municipal corporation carried out information, education and communication activities in the affected areas to inform the residents of preventive measures like chlorination of drinking water, awareness generation on hand washing with soap and water for everyone after defecation and before preparing/serving/eating food, (ii) to create a proper drainage system in order to avoid stagnation of waste water, (iii) to boil the drinking water, (iv) not to buy antibiotics over the counter without consulting a physician for any diarrhoea episode in future. These measures will help to improve hygiene and infection control practices, which are needed for the prevention of diarrhoea.
